# Perinatal western‐type diet and associated gestational weight gain alter postpartum maternal mood

**DOI:** 10.1002/brb3.828

**Published:** 2017-09-14

**Authors:** Jessica L. Bolton, Melanie G. Wiley, Bailey Ryan, Samantha Truong, Melva Strait, Dana Creighton Baker, Nancy Y. Yang, Olga Ilkayeva, Thomas M. O'Connell, Shelley W. Wroth, Cristina L. Sánchez, Geeta Swamy, Christopher Newgard, Cynthia Kuhn, Staci D. Bilbo, Leigh Ann Simmons

**Affiliations:** ^1^ Department of Psychology and Neuroscience Duke University Durham NC USA; ^2^ Duke Integrative Medicine Durham NC USA; ^3^ Department of Medicine Duke University School of Medicine Durham NC USA; ^4^ Duke University School of Nursing Durham NC USA; ^5^ Duke Molecular Physiology Institute Duke University School of Medicine Durham NC USA; ^6^ Department of Pharmacology and Cancer Biology Duke University School of Medicine Durham NC USA; ^7^ Department of Obstetrics and Gynecology Duke University School of Medicine Durham NC USA

**Keywords:** dopamine, gestational weight gain, histidine, postpartum anxiety branched‐chain amino acids, postpartum depression

## Abstract

**Introduction:**

The role of perinatal diet in postpartum maternal mood disorders, including depression and anxiety, remains unclear. We investigated whether perinatal consumption of a Western‐type diet (high in fat and branched‐chain amino acids [BCAA]) and associated gestational weight gain (GWG) cause serotonin dysregulation in the central nervous system (CNS), resulting in postpartum depression and anxiety (PPD/A).

**Methods:**

Mouse dams were fed one of four diets (high‐fat/high BCAA, low‐fat/high BCAA, high‐fat, and low‐fat) prior to mating and throughout gestation and lactation. Postpartum behavioral assessments were conducted, and plasma and brain tissues assayed. To evaluate potential clinical utility, we conducted preliminary human studies using data from an extant sample of 17 primiparous women with high GWG, comparing across self‐reported postpartum mood symptoms using the Edinburgh Postnatal Depression Scale (EPDS) for percent GWG and plasma amino acid levels.

**Results:**

Mouse dams fed the high‐fat/high BCAA diet gained more weight per kcal consumed, and BCAA‐supplemented dams lost weight more slowly postpartum. Dams on BCAA‐supplemented diets exhibited increased PPD/A‐like behavior, decreased dopaminergic function, and decreased plasma tyrosine and histidine levels when assessed on postnatal day (P)8. Preliminary human data showed that GWG accounted for 29% of the variance in EPDS scores. Histidine was also lower in women with higher EPDS scores.

**Conclusions:**

These findings highlight the role of perinatal diet and excess GWG in the development of postpartum mood disorders.

## 1 INTRODUCTION

Postpartum depression and anxiety (PPD/A) are serious and highly prevalent complications of pregnancy. Data show that upward of 20% of parturient women suffer from a postpartum mood disorder with 7.1% of postpartum women experiencing a major depressive disorder, up to 19.2% experiencing any depression within 3 months of childbirth, and 8–12% of postpartum women experiencing an anxiety disorder, including generalized anxiety and obsessive‐compulsive disorder (Fairbrother, Janssen, Antony, Tucker, & Young, 2016; Gavin et al., 2005). Despite the many well‐documented negative effects of PPD/A on both maternal (Eaton, Armenian, Gallo, Pratt, & Ford, 1996; Joynt, Whellan, & O'Connor, 2003; Musselman, Evans, & Nemeroff, 1998; Norhayati, Hazlina, Asrenee, & Emilin, 2015; O'Hara & McCabe, 2013; Paul, Downs, Schaefer, Beiler, & Weisman, 2013; Yim, Tanner Stapleton, Guardino, Hahn‐Holbrook, & Dunkel Schetter, 2015) and child (Ashman, Dawson, & Panagiotides, 2008; Dawson et al., 2003; Elgar, McGrath, Waschbusch, Stewart, & Curtis, 2004; Glasheen, Richardson, & Fabio, 2010; Goodman & Brand, 2008; Goodman & Tully, 2007; Lundy & Field Jeffrey, 1996; Marchand & Hock, 1998; Stein et al., 2014; Zahn‐Waxler, Iannotti, Cummings, & Denham, 1990) health, the biological and psychosocial mechanisms through which PPD/A develop remain unclear (Deecher, Andree, Sloan, & Schechter, 2008; Pawluski, Lonstein, & Fleming, 2017; Payne, Palmer, & Joffe, 2009; Zonana & Gorman, 2005). Complicating our understanding of these mood disorders is the fact that PPD/A often occur comorbidly, with PPA frequently preceding PPD (Falah‐Hassani, Shiri, & Dennis, 2016; Reck et al., 2008), and there is growing consensus that anxiety may be a feature of PPD (Navarro et al., 2007). Preventive psychological and psychosocial interventions have had limited success in reducing the incidence of PPD/A, thus providing few clues about root causes. Mechanistic theories for the etiology of PPD/A also range widely. Studies suggest that PPD may result from: (a) inability of the hypothalamic–pituitary–adrenal (HPA) axis to maintain homeostasis when exposed to challenges like pregnancy and parturition (Jolley, Elmore, Barnard, & Carr, 2007); (b) postpartum hormonal withdrawal from high prenatal levels of progesterone and estrogen (Corwin & Pajer, 2008; Deecher et al., 2008); and (c) immune activation associated with pregnancy and parturition (Maes, Ombelet, Verkerk, Bosmans, & Scharpe, 2001; Maes et al., 2002). Even less is known about the biological etiology of PPA, but research has identified some significant biological correlates of PPA that overlap with PPD, including levels of estrogen, progesterone, adrenal corticosteroids, prolactin, oxytocin, norepinephrine, and serotonin (Lonstein, Maguire, Meinlschmidt, & Neumann, 2014). These diverse findings suggest additional biobehavioral contributors to PPD/A yet to be identified.

One novel area of research that may advance our understanding of women's vulnerability to PPD/A is the effect of perinatal diet on the generation of neurotransmitters that regulate mood. More than one third of pregnant women consume a primarily Western diet, defined as overconsumption of animal protein, refined carbohydrates, and fatty foods (Englund‐Ogge et al., 2014). These diets are overabundant in branched‐chain amino acids (BCAA), found mostly in red meats but also in pork, poultry, eggs, and fish. BCAA include leucine (Leu), isoleucine (Ile), and valine (Val), and are three of the nine essential amino acids that must be obtained exogenously via the food supply (Harper, Miller, & Block, 1984). The BCAA are also important during pregnancy and postpartum recovery. These states induce significant metabolic changes, including protein accretion, that are designed to synthesize new maternal and fetal tissues, ensure the fetus receives a continuous supply of nutrients despite intermittent maternal food intake, and support lactation after birth (Kalhan, 2000; Kalhan & Parimi, 2006; Kalhan, Rossi, Gruca, Super, & Savin, 1998). During healthy pregnancy there is a downregulation of the rate of BCAA transamination, especially for leucine, resulting in higher plasma and extracellular fluid levels of BCAA as compared to the nonpregnant state (Jolly et al., 2004; Kalhan and Parimi). When there is excess BCAA consumption as is typical in the Western‐type diet, this increases the BCAA load on an already downregulated system, resulting in lower rates of protein turnover and higher whole‐body levels of BCAA (Jolly et al., 2004; Kalhan & Parimi, 2006).

We hypothesize that perinatal consumption of a Western diet increases risk for PPD/A by increasing concentrations of plasma BCAA, causing competition for transport of all large neutral amino acids across the blood–brain barrier into the central nervous system (CNS) via the **L**arge neutral **A**mino acid **T**ransporter 1 (LAT‐1) (Fernstrom, 2005). This includes transport of BCAA *as well as* the amino acids histidine (HIS), tryptophan (TRP), and tyrosine (TYR), which are precursors to key neurotransmitters implicated in PPD/A (Nutt, 2001; Senkowski, Linden, Zubrägel, Bär, & Gallinat, 2003; Werner & Coveñas, 2010) (i.e., histamine, serotonin, dopamine, and norepinephrine). An increase in one of the large neutral amino acids induces decreased uptake of the others, and vice versa (Fernstrom, 2005). Furthermore, the rate of production of these neurotransmitters depends on the availability of their amino acid precursors (Wurtman & Fernstrom, 1975; Young & Gauthier, 1981).

The primary aim of this study was to use a mouse model to investigate the hypothesis that perinatal consumption of a diet high in fat and BCAA, as in a Western‐type diet, and associated gestational weight gain (GWG) cause serotonin dysregulation in the CNS resulting in postpartum depressive‐like and anxiety‐like behavior. The secondary aim was to determine whether we could establish preliminary clinical evidence in a small cohort of mothers for the relationships among diet, GWG, BCAA concentrations, and postpartum mood identified in the animal studies. This work is clinically relevant because diet is a modifiable behavior that is safe to manipulate during pregnancy when based on principles of sound nutrition.

## 2 MATERIALS AND METHODS

### 2.1 Animal studies

#### 2.1.1 Animals

All procedures were approved by the Duke University Institutional Animal Care and Use Committee. Juvenile (P28) female C57BL/6 mice were obtained from Charles River Laboratories (Raleigh, NC) in three separate cohorts (*n *= 189) and housed in individually ventilated cages with ad libitum access to food (PicoLab Mouse Diet 5058, Lab‐Diet, Philadelphia, Pennsylvania) and filtered water. Adult breeder males were placed with 2–3 females each for breeding, for a maximum of 3 weeks. The colony was maintained at 22°C on a 12:12‐h light‐dark cycle (lights on at 7 AM). Sentinel animals were housed in the colony room and screened periodically for the presence of common rodent diseases; all screens were negative.

#### 2.1.2 Diets

On P35‐P40, females were randomly placed on one of four customized diets (Research Diets, New Brunswick, NJ, USA): a low‐fat diet [LFD/Control, comparable to a normal rodent chow diet; 19% of calories from protein, 71% from carbohydrate, and 10% from fat (56:44% soybean oil:lard); cat. no. D07010502; *n *=* *35], a high‐fat diet [HFD/Control; 20% of calories from protein, 35% from carbohydrate, and 45% from fat (12:88% soybean oil:lard); cat. no. D12451; *n *=* *59], a low‐fat diet supplemented 150% with leucine, isoleucine, and valine (BCAA) [LFD/BCAA; 23% of calories from protein, 67% from carbohydrate, and 10% from fat (56:44% soybean oil:lard); cat. no. D07010503; *n *=* *35], and a high‐fat diet supplemented 150% with leucine, isoleucine, and valine [HFD/BCAA; 23% of calories from protein, 34% from carbohydrate, and 43% from fat (12:88% soybean oil:lard); cat. no. D06050807; *n *=* *60] (Coppola et al., 2013). The HFD and HFD/BCAA diets provided 4.7 kcal/g; the LFD provided 3.84 kcal/g; and the LFD/BCAA provided 3.85 kcal/g. The complete nutritional profiles of each diet are available online (http://www.researchdiets.com). Females were weighed prior to diet assignment (baseline weights did not differ significantly by diet group), and every 3 days thereafter until parturition (the final peripartum weight was thus collected between E16 and E18, see Figure 2d). Food intake was measured by weighing the food every 3 days and dividing the change in food weight by the number of females in the cage. After parturition, to minimize disruption when dams were alone with their litters, maternal weights and food consumption were recorded on P10, P14, P21, and P28.

#### 2.1.3 Experimental timeline

**Figure 1:**
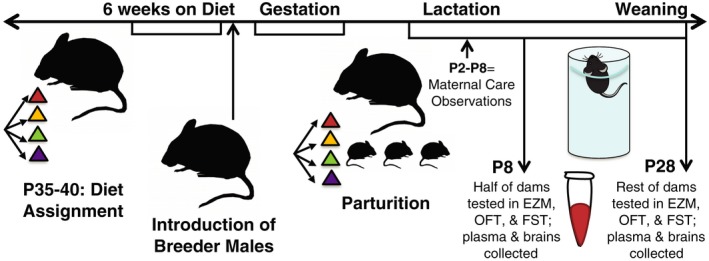
Experimental timeline for animal studies. P35‐P40 female mice were placed on one of four diets (LFD/Control, HFD/Control, LFD/BCAA, or HFD/BCAA) and fed ad libitum for 6 weeks before the introduction of adult breeder males. Dams (*n *=* *16–20/diet group) were singly housed once visibly pregnant, were allowed to give birth naturally, and remained on the diets throughout pregnancy and lactation. All litters were observed and maternal behavior scored three times daily from P2 to P8. Eight days after parturition, half of the dams (*n *=* *8–11/diet group) underwent behavioral assessments in elevated zero maze (EZM), open field test (OFT), and Porsolt forced‐swim test (FST), blood draws, and sacrifice to obtain brain tissue for analysis. For the remaining dams (*n *=* *8–9/diet group), pups were weaned on P28, and immediately afterward dams underwent behavioral assessments, blood draws, and sacrifice for brain tissue analysis as at P8

Females were fed ad libitum for 6 weeks before breeding and remained on the diets throughout pregnancy and lactation. Dams (*n *=* *16–20/diet group) were singly housed once visibly pregnant, and allowed to give birth naturally. All litters were observed and maternal behavior scored three times daily from P2 to P8. On P8, half of dams (*n *=* *8–11/diet) underwent behavioral assessments in elevated zero maze (EZM) (Shepherd, Grewal, Fletcher, Bill, & Dourish, 1994), open field test (OFT) (Gould, Dao, & Kovacsics, 2009), and Porsolt forced‐swim test (FST) (Castagné, Moser, Roux, & Porsolt, 2011), blood draws, and sacrifice to obtain brain tissue for analysis. For remaining dams (*n *=* *8–9/diet), pups were weaned on P28, and immediately afterward dams underwent behavioral assessments, blood draws, and sacrifice for brain tissue analysis (see Figure 1 for timeline schematic). Females not pregnant after 3 weeks with males were tested after 13 weeks on diet (to mirror the time pregnant females remained on diet prior to sacrifice) in EZM, OFT, and FST, and sacrificed to collect blood and brain samples for later analysis (*n *=* *6–15/group; pregnancy rates varied nonsignificantly by group).

### 2.2 Behavioral assessments

#### 2.2.1 Maternal care

Maternal care was assessed on days P2–P8 for 3 hr each day, twice during the light phase (0900–1000, 1500–1600 hr) and once during the dark phase (2100–2200 hr). Raters (*n *= 5) trained by an experienced (Löwe, Kroenke, Herzog, & Gräfe, 2004) coder to >95% reliability and blinded to experimental condition live scored the instantaneous occurrence (frequency) of the following behaviors every 5 min for the duration of the hour: being on the nest, arch‐backed nursing, blanket nursing (lying down with little to no arch in the back to nurse pups), passive nursing (lying on the side to nurse pups), licking and grooming pups, self‐grooming, eating, or nest building. For each behavior, the percentage of observations that the dam was performing each of the behaviors during each observation session (number of times observed / 20 total observations per hour × 100) was calculated, averaged over days and other observation sessions in day versus night, and analyzed.

#### 2.2.2 Elevated zero maze

Elevated zero maze is a widely used test for anxiety‐like behavior in rodents (Bradburn, Frankel, Baker, & Pergamit, 1991). The maze has an elevated (49.5 cm high) circular lane (4.5 cm wide) divided into four quadrants. Two opposite quadrants are enclosed by walls (15.9 cm high), whereas the remaining two quadrants are left exposed. Each mouse was placed onto the interface between an open and closed arm, and live scored for total time spent in the open arms out of 5 min, as well as number of stretch‐attend postures from a closed to open arm. A decrease in time spent in the open arms is indicative of increased anxiety. The maze was cleaned between animals.

#### 2.2.3 Open field test

Open field test was used to assess activity levels and anxiety‐like behavior (Schauder, Zavelberg, Langer, & Herbertz, 1987). Animals were placed in a square enclosure 40 cm by 45.1 cm with walls 34.9 cm high and allowed to explore freely for 10 min. Total distance traveled, mean speed, and time mobile were used as metrics of overall activity, while time in the surround (the area closest to the walls of the maze, ~50% of total area) and percent of distance traveled in the surround were used to quantify anxiety‐like behavior. All behaviors were recorded using ANY‐maze video‐tracking software (Stoelting Co., Wood Dale, IL, USA).

#### 2.2.4 Forced‐swim test

Forced‐swim test is a widely used test for depressive‐like behaviors in rodents (Castagné et al., 2011) that was selected to preclude the changes to dietary intake inherent with other tests, such as sucrose consumption, and the dangers associated with the tail suspension task in accommodating high body weights due to diet and pregnancy. The apparatus is a Plexiglas cylinder (20.3 cm diameter × 49.5 cm high) filled two third full with 22°C water. Each mouse was lowered into the center of the water‐filled cylinder, and then scored for the duration of immobility for 6 min. Immobility was defined as the absence of all swimming, except for the subtle motions required to keep the head above water. In our tests, immobility time was not observed to be dependent on the body weight of the animal. This test is stressful for the animals; therefore, it was conducted last on the day of sacrifice to avoid unwanted behavioral effects in other tests. Animals were sacrificed within 3–5 min of the end of the forced‐swim test to minimize the effects of stress on brain neurochemistry.

#### 2.2.5 Blood and brain collection

Blood (100–150 μl) was drawn via the facial maxillary gland using a 5‐mm lancet and collected into microcentrifuge tubes containing EDTA. Whole‐blood glucose was assessed using a glucometer (TRUE2go; CVS Pharmacy, Inc., Woonsocket, RI, USA). Mice were then anesthetized via ketamine/xylazine (430 mg/kg ketamine; 65 mg/kg xylazine, i.p.), and transcardially perfused with ice‐cold saline for 2 min. Whole brains were rapidly extracted and dissected into hippocampus (HIPP; cut into two halves) and prefrontal cortex (PFC), and snap frozen in liquid nitrogen. Blood was centrifuged at 1,200 *g* at 4°C for 10 minutes, and plasma was removed and stored along with brain tissue at −80°C until assayed.

#### 2.2.6 qRT‐PCR

Total RNA was isolated from half‐HIPP using the Protein and RNA Isolation System (PARIS kit; Ambion, Waltham, MA, USA) according to manufacturer's instructions. cDNA was synthesized from 100 ng of RNA using the QuantiTect reverse transcription kit (Qiagen, Inc., Valencia, CA, USA), and quantitative real‐time PCR (qRT‐PCR) was performed using a QuantiFast SYBR Green PCR kit (Qiagen) on a Mastercycler ep realplex (Eppendorf, Hauppauge, NY, USA) as previously described (Williamson, Sholar, Mistry, Smith, & Bilbo, 2011). Briefly, 1 μl of cDNA was added to 12 μl of master mix containing specific primers for genes of interest. We designed primer pairs for all genes (*Gapdh*,* Htr1a*,* Htr2c*,* Slc6a4*,* Drd1*, and *Drd2*; see Table 1) as previously described (Tai et al., 2010). Designed primers were obtained from Integrated DNA Technologies, Inc. (Coralville, IA, USA). Optimal annealing temperatures for each primer pair were determined by running a temperature gradient, and specificity was verified by melt‐curve analysis. For analysis, we determined the threshold cycle (CT) for each reaction and calculated relative gene expression using the 2^−ΔΔCt^ method (Livak & Schmittgen, 2001; Rao, Huang, Zhou, & Lin, 2013; Tai et al., 2010), using *Gapdh* as a reference gene.

**Table 1:** PCR primers obtained from Integrated DNA Technologies, Inc. Optimal annealing temperatures for each primer pair were determined by running a temperature gradient, and specificity was verified by melt‐curve analysis

Gene name	Forward primer sequence	Reverse primer sequence
*Gapdh*	ggtcaccagggctgccattt	tgggcttcccgttgatgaca
*Htr1a*	ctgccgctgatgatgatgatg	gagtgaacaggaagggtcc
*Htr2c*	cgatggtggacgcttgtttc	gataacgagaatgttgcccc
*Slc6a4*	gctgagatgaggaacgaag	ggcaaagaatgtggatgctg
*Drd1*	gagtgattgggggaagtc	gacaggataagcagggacag
*Drd2*	cctccatcgtctcgttctac	gagtggtgtcttcaggttgg
*Slc6a3*	cttcactgtcatcctcatc	gtcccaaaggtgtcgttg

#### 2.2.7 High‐performance liquid chromatography with electrochemical detection (HPLC‐EC)

Two hundred and fifty microliters of ice‐cold standard buffer (0.5 mmol/L sodium metabisulfite, 0.2 N perchloric acid, and 0.5 mmol/L EDTA) was added to thawed half‐HIPP or PFC. The tissue was disrupted by sonication until completely homogenized, then centrifuged at 16,000 *g* for 10 min at 4°C. The supernatant was collected and filtered through a 0.45‐μm membrane via centrifugation (Durapore PVDF centrifugal filters, Millipore, Billerica, MA, USA), and kept on ice until analysis for the amino acid tryptophan (Trp), norepinephrine (NE), indoleamines (5‐HT and 5‐HIAA), and dopamine and its respective metabolites (DA, DOPA, and HVA) as previously described (Sánchez, Van Swearingen, Arrant, Kuhn, & Zepf, 2014). Processed samples were separated using a 100 × 4.6 mm Kinetex (C18 5 μm 100 Å, Phenomenex) column on a reverse‐phase HPLC system with a BAS LC‐4B electrochemical detector with dual 3‐mm carbon electrode (MF‐1000) and reference electrode (MF‐2021) as previously described (Brotman et al., 2008). An external standard curve of all analytes was run each day. Trp quantification was performed using a mobile phase consisting of 8% acetonitrile (v/v), 0.05 mol/L citric acid, 0.05 mol/L Na_2_HPO_4_•7H2O, and 0.1 mmol/L EDTA. No correction for pH was needed. The detector was set to 0.875 V versus Ag/AgCl reference electrode, sensitivity at 20 nA, and a flow rate of 1.0 ml/min. Indoleamines and catecholamines were separated with a mobile phase consisting of 18% methanol (v/v), 0.1 mol/L sodium phosphate, 0.8 mmol/L octanesulfonic acid (anhydrous), and 0.1 mmol/L EDTA (final pH adjusted to 3.1). The detector was set to 0.70 V, sensitivity at 20 nA, and a flow rate of 1.0 ml/min.

### 2.3 Preliminary human studies

#### 2.3.1 Human subjects

The Duke University Institutional Review Board approved all procedures. Data were analyzed from 17 women who participated in a pilot randomized trial examining health coaching for women who exceeded Institute of Medicine (IOM) recommendations for GWG, based on prepregnancy body mass index (BMI). Eligible participants included primiparous women between 18 and 45 years of age with full‐term, uncomplicated, singleton pregnancies who were recruited within 24 hr of delivery from the birthing centers at Duke Regional Hospital and the Duke University Medical Center. Exclusion criteria included current or recent (within the last 6 months) smoker, inability to read and understand English, and complications of delivery that would increase risk of blood draw (e.g., dehydration, anemia). Data included for the present analysis were obtained at baseline via the electronic medical record or at 4–6 weeks postpartum via standardized self‐report instruments using a computer‐assisted self‐interview (CASI) prior to participation in any intervention procedures.

#### 2.3.2 Blood samples

A fasting (≥8 hr) 60‐ml blood sample was obtained by antecubetal venipuncture within 48 hr of delivery and prior to hospital discharge. Samples were drawn into chilled EDTA‐treated vacutainers, immediately transferred on ice to the laboratory, and spun for 10 min at 1200 *g* using a balanced, refrigerated (4^o^ C) centrifuge. The plasma, sera, and buffy coats were then transferred to 1.7‐ml polypropylene microcentrifuge tubes and stored at −70°C until assayed.

#### 2.3.3 Postpartum mood

Approximately 1 month after delivery (M = 31 days), women participated in an interview including an assessment of postpartum mood using the Edinburgh Postnatal Depression Scale (EPDS) (Cox, Holden, & Sagovsky, 1987; Eberhard‐Gran, Eskild, Tambs, Opjordsmoen, & Samuelsen, 2001). The EPDS is a 10‐item, self‐reported scale that has been validated in diverse populations of pregnant and postpartum women for identifying women at risk for PPD/A (Brouwers, van Baar, & Pop, 2001; Ross, Evans, Sellers, & Romach, 2003; Ross, Sellers, Gilbert Evans, & Romach, 2004). Specifically, the EPDS includes measurement of symptoms of both depression and anxiety, and total scores have been validated to identify risk for either disorder (Brouwers et al., 2001). In this study, a cut‐off score of 10 was used for the EPDS, which has reported sensitivities and specificities of 70–90% in multiple studies, is between the high (e.g., 12, 13) and low (e.g., 6, 7, 8) cut‐off scores used previously (Cox et al., 1987; Eberhard‐Gran et al., 2001), and is a recommended cut‐off score for psychiatric referral (Eberhard‐Gran et al., 2001). Given the exploratory nature of the study, we have tolerated some false positives, recognizing that scores in the intermediate range indicate clinically significant symptoms of PPD/A.

#### 2.3.4 Gestational weight gain (GWG)

GWG was calculated as the percent change in BMI (%ΔBMI) from prepregnancy to last recorded pregnant weight (occurring no more than 1 week prior to delivery). Data were abstracted from the medical record to obtain BMI, calculated using the standard formula (weight [kg] / height [m^2^]).

#### 2.3.5 Plasma amino acid measurement

Plasma amino acids were measured by flow injection tandem mass spectrometry using stable isotope dilution techniques as described previously (Haqq et al., 2005; Laferrère et al., 2011; Lien et al., 2009; Newgard et al., 2009). Data were acquired using a Waters triple quadrupole detector equipped with Acquity UPLC system and controlled by the MassLynx 4.1 MS software platform (Waters, Milford, MA).

### 2.4 Data analysis and statistics

All data were collected without knowledge of treatment group and analyzed using SPSS statistical software (IBM, Armonk, NY, USA). All animal measures were analyzed using two‐way (HFD X BCAA) ANOVAs. If heterogeneous variance was noted between groups, outliers were first identified and removed via Tukey's IQR method. Following significant *F* scores for interactions, post hoc comparisons (Tukey's HSD) were performed to further distinguish among groups, and all differences were considered statistically significant if *p *<* *.05. Descriptive statistics were calculated for human subjects’ age, EPDS scores, and percent change in BMI from prepregnancy to delivery. A simple correlation and coefficient of determination were calculated relating BMI change to EPDS score. Plasma amino acids in human samples were analyzed via nonparametric Wilcoxon rank‐sum tests (also known as Mann–Whitney *U* tests), due to the disparity in sample size between the groups.

## 3 RESULTS

### 3.1 Diets high in fat and BCAA generate an obese phenotype in female mice

**Figure 2:**
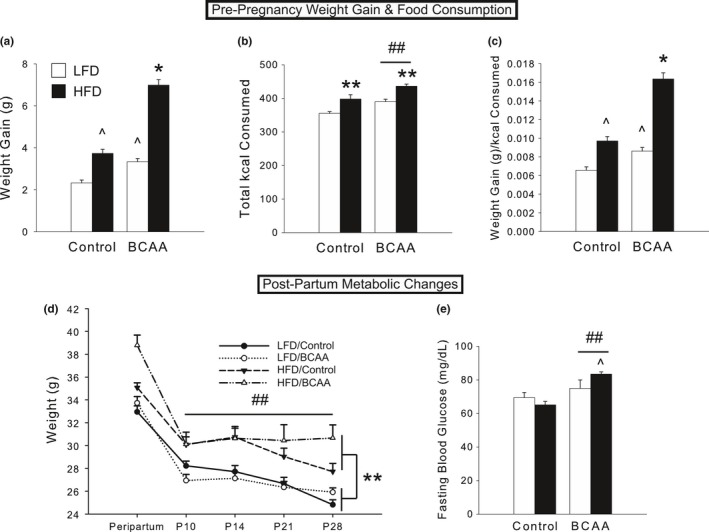
Diets high in fat and BCAA generate an obese phenotype in female mice. (a) After 6 weeks on diet but prior to breeding, body weight was increased in both HFD and BCAA groups, with a synergistic effect in HFD/BCAA females. (b) HFD and BCAA groups consumed more kilocalories than controls. (c) HFD/BCAA dams gained more weight than other groups, even after controlling for kilocalories of food consumed. (d) Following parturition, BCAA‐supplemented dams lost weight at a slower rate in the postpartum period from P10 to P28. HFD did not affect postpartum weight loss rate, but HFD dams remained significantly heavier than LFD dams. (e) BCAA supplementation, and particularly the combination of HFD and BCAA, resulted in increased fasting blood glucose at P8. For a–c, data are mean ± SEM,* n *=* *35–60 animals/group for weight gain; *n *=* *15–21 cages/group for food consumption. For d, data are mean ± SEM,* n *=* *8–9 animals/group. For e, data are mean ± SEM,* n *=* *8–11animals/group. *p <* *.001 versus all other groups; **p <* *.001, HFD versus LFD; ^##^ *p *<* *.005, BCAA versus Control

Both HFD and BCAA animals gained significantly more weight than controls by week 6 on the diet, with a synergistic effect in the HFD/BCAA group [significant HFD X BCAA interaction, *F*(1,185)  = 24.39, *p *<* *.001; post hoc, *p *<* *.05; Figure 2a]. During this period, HFD and BCAA groups consumed more kilocalories than the control group [significant main effects of HFD, *F*(1,68)  = 22.75, *p *<* *.001; and BCAA, *F*(1,68)  = 15.63, *p *<* *.001; Figure 2b], likely due to the greater palatability and/or energy density of these diets (Coppola et al., 2013). However, HFD/BCAA animals gained significantly more weight per kilocalorie consumed than did all other diet groups over the 6 weeks on diet [significant HFD X BCAA interaction, *F*(1,185)  = 16.84, *p *<* *.001; post hoc, *p *<* *.05; Figure 2c], suggesting an effect of diet on metabolic regulation, or digestion efficiency/absorption. No difference was observed between diets in percent weight gained or food consumed during pregnancy, and HFD/BCAA animals remained heavier than all other groups (data not shown).

Between the peripartum period and weaning, when maternal body weight typically declines as a consequence of nursing, BCAA‐supplemented dams exhibited a markedly slower rate of weight loss compared to other groups [repeated measures ANOVA, significant Time x BCAA interaction, *F*(4,136)  = 4.18, *p *<* *.005; Figure 2d]. HFD did not alter the rate of postpartum weight loss, but HFD dams remained significantly heavier overall due to their higher weight before parturition [between‐subjects main effect of HFD, *F*(1,32)  = 25.98, *p *<* *.001]. In addition, BCAA‐supplemented dams had significantly elevated fasting blood glucose levels at P8 [significant main effect of BCAA, *F*(1,27)  = 14.95, *p *<* *.001], with HFD/BCAA dams exhibiting the highest levels [significant HFD X BCAA interaction, *F*(1,27)  = 4.33, *p *<* *.05; Figure 2e]. This effect disappeared by P28 (data not shown).

### 3.2 BCAA‐supplemented diets induce a PPD‐like phenotype, and diets high in fat induce anxiety‐like behavior

**Figure 3:**
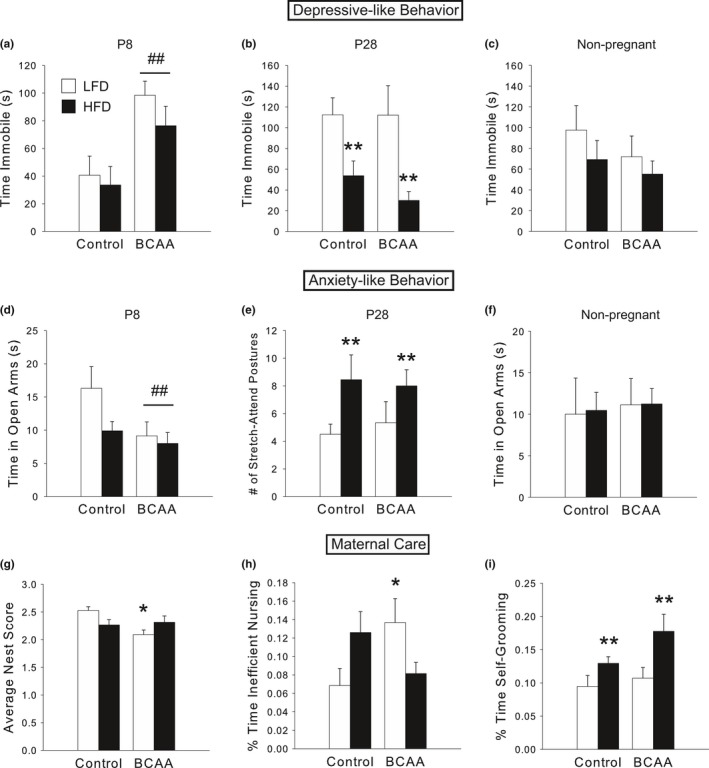
BCAA supplementation induces a postpartum depression‐like phenotype, and diets high in fat result in postpartum anxiety‐like behavior. (a) At P8, BCAA dams were more immobile in the FST than controls. (b) At P28, HFD dams were less immobile than LFD dams. (c) These PPD‐like effects were dependent on pregnancy, as nonpregnant females on the diets exhibited no significant differences. (d) At P8, BCAA dams spent less time in the open arms than controls, and HFD dams tended to show a similar pattern. (e) At P28, HFD dams performed more stretch‐attend postures in the EZM, which are characteristic of increased anxiety‐like behavior, than LFD controls. (f) Nonpregnant females did not exhibit significant differences in anxiety‐like behavior due to diet. (g) Postpartum BCAA dams, especially LFD/BCAA dams, had nests of lower quality than controls. (h) LFD/BCAA dams spent more of their total time nursing during the light cycle (when most nursing occurs) in inefficient or passive postures (i.e., blanket and passive nursing as opposed to arched‐back nursing). (i) HFD dams spent more time self‐grooming during the dark cycle than LFD controls, which may be indicative of anxiety‐like behavior. For a–f, data are mean ± SEM,* n *=* *7–10/group. For g–i, data are mean ± SEM,* n *=* *17–20/group (note that dams sacrificed at P8 and P28 are combined here). ^##^ *p *<* *.05, BCAA versus Control; **p <* *.05, HFD versus LFD; *p <* *.05 versus LFD/Control

At 8 days postpartum, BCAA dams spent more time immobile in the FST than controls [main effect of BCAA, *F*(1,30)  = 14.64, *p *<* *.005; Figure 3a], an effect which disappeared by the time pups were weaned at P28 (Figure 3b), indicating a depressive‐like response during the postpartum period. Furthermore, BCAA dams spent less time exploring the open arms of the EZM specifically at P8 compared to controls [main effect of BCAA, *F*(1,29)  = 4.69, *p *<* *.05; Figure 3d], suggesting increased postpartum anxiety‐like behavior as well. Importantly, nonpregnant BCAA animals did not differ from controls (Figure 3c,f), indicating the effects of BCAA on behavior were dependent on pregnancy. An alternative explanation of this data is that pregnancy and parturition decreased anxiety primarily in the LFD/Control dams, as has been previously reported for normal postpartum rodents (Lonstein, 2005), but did not have an anxiolytic effect on the BCAA groups. Neither of these effects can be explained by a BCAA‐induced decrease in general locomotor activity (data not shown).

HFD dams exhibited significantly more stretch‐attend postures on P28 [significant main effect of HFD, *F*(1,34)  = 5.33, *p *<* *.05; Figure 3e], which is indicative of increased anxiety‐like behavior (although time in the open arms at P28 did not differ significantly among groups). In addition, P28 HFD dams spent less time immobile in the FST (Figure 3b), which may also indicate elevated anxiety (Detke, Rickels, & Lucki, 1995; Heisler et al., 1998). Together, these data suggest that HFD‐induced anxiety is not restricted to the early postpartum period, as was the case for the BCAA effect.

### 3.3 HFD and BCAA dams exhibit altered maternal care behaviors

During the critical first postnatal week, LFD/BCAA dams had lower nest scores relative to control dams [based on a nest quality score of 0 to 3 performed by observers blind to experimental group; significant HFD X BCAA interaction, *F*(1,62)  = 7.05, *p *<* *.05; post hoc, *p *<* *.05; Figure 3g]. Moreover, during the light cycle, when dams spend most of their time nursing, LFD/BCAA moms spent a higher percentage of their time nursing their pups in passive, inefficient nursing postures (i.e., blanket and passive nursing, as opposed to arched‐back nursing) compared to LFD/Control moms [significant HFD X BCAA interaction, *F*(1,63)  = 7.18, *p *<* *.01; post hoc, *p *<* *.05; Figure 3h]. These data may be consistent with the postpartum depressive‐like phenotype observed in the behavioral tests.

HFD dams spent more time during the dark cycle self‐grooming [significant main effect of HFD, *F*(1,63)  = 9.83, *p *<* *.005; Figure 3i]. Given the finding of increased anxiety‐like behavior in postpartum HFD dams, it is possible that this excessive self‐grooming represents a repetitive, anxious phenotype (Shmelkov et al., 2010; Welch et al., 2007).

**Figure 4:**
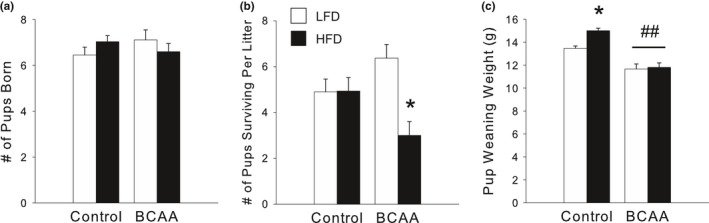
(a) Litter size at birth did not differ by perinatal diet. (b) HFD/BCAA dams had fewer pups survive to weaning at P28 than all other diet groups. (c) Offspring of BCAA‐supplemented dams were significantly smaller at weaning than offspring of Controls. Offspring of HFD/Control dams, unlike those of HFD/BCAA dams, were significantly larger than offspring of LFD/Control dams at weaning. Data for a and b are mean ± SEM,* n *=* *19–32 litters/group. Data for c are mean ± SEM,* n *=* *8–9 litters/group. *p <* *.05 versus all other groups; ^##^ *p *<* *.05, BCAA versus Controls

Consistent with the observed changes in maternal depression‐ and anxiety‐like behaviors, as well as maternal care behaviors, due to HFD and BCAA, HFD/BCAA dams had fewer pups survive to weaning than all other groups [significant HFD X BCAA interaction, *F*(1,99)  = 7.34, *p *<* *.01; post hoc, *p *<* *.05; Figure 4b], despite equal litter sizes at birth (Figure 4a), as HFD/BCAA dams tended to neglect or cannibalize their pups in early life. Furthermore, offspring born to BCAA dams were significantly smaller than controls at weaning [litter average weights; significant main effect of BCAA, *F*(1,32)  = 56.75, *p *<* *.001; Figure 4c]. Together, the above results demonstrate an effect of HFD and BCAA on maternal behavior during a critical period of neonatal development.

### 3.4 Diets high in fat and BCAA alter postpartum monoamine neurochemistry

**Figure 5:**
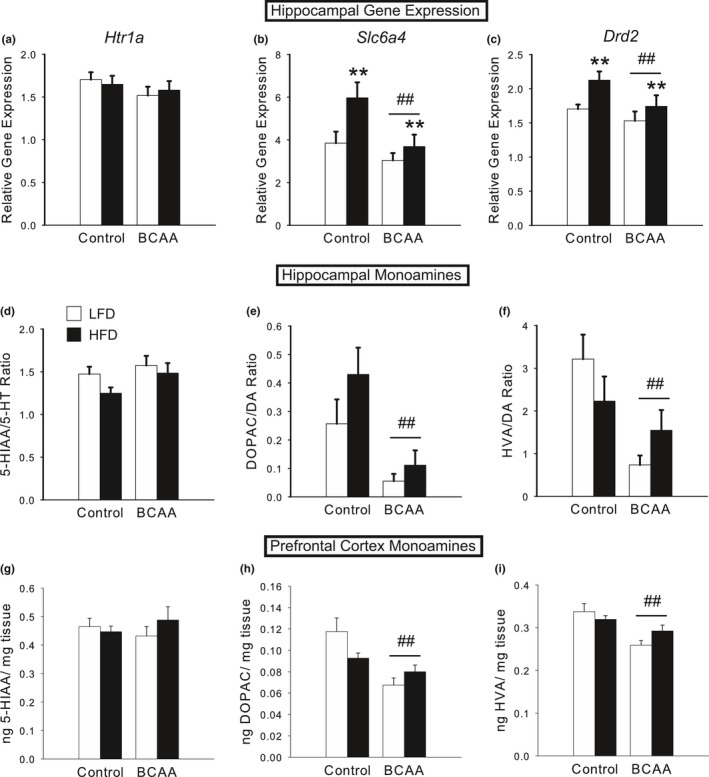
Diets high in fat and BCAA alter postpartum monoamine neurochemistry. (a) Expression of *Htr1a*, the gene that encodes the 5‐HT1a serotonin receptor, did not change due to diet in the hippocampus of P8 dams. (b) Expression of *Slc6a4*, which encodes the 5‐HTT serotonin transporter, was significantly decreased in the hippocampus of P8 BCAA dams, but increased by HFD. (c) Expression of *Drd2*, the gene that encodes the D2 dopamine receptor, was significantly decreased in the hippocampus of BCAA dams, but increased by HFD at P8. (d) HPLC‐EC analysis of monoamine neurotransmitter levels in the hippocampus of P8 dams revealed no significant differences due to diet in serotonin turnover rate. (e–f) However, BCAA supplementation induced marked decreases in dopamine turnover rates in the hippocampus at P8, as represented by the DOPAC/DA ratio (e) and HVA/DA ratio (f). (g–i) HPLC‐EC analysis of monoamine neurotransmitter levels in the prefrontal cortex of P8 dams revealed no significant differences due to diet in levels of the serotonin metabolite 5‐HIAA (g), but marked differences in levels of the dopamine metabolites DOPAC (h) and HVA (i), just as in the hippocampus of P8 dams. Data are mean ± SEM,* n *=* *7–11/group. ^##^ *p *<* *.05, BCAA versus Control; **p <* *.05, HFD versus LFD

Due to our observation of a PPD‐like phenotype in BCAA mothers, we assessed the hippocampus (a limbic region critical for mood regulation) for alterations in monoamine receptors and transporters using qRT‐PCR. P8 BCAA dams had significantly decreased serotonin transporter expression [significant main effect of BCAA, *F*(1,30)  = 5.71, *p *<* *.05; Figure 5b], but no significant changes in the expression of two serotonin receptor subtypes (5‐HT1a in Figure 5a; 5‐HT2c not shown). Dopamine D2 receptor was also significantly decreased [significant main effect of BCAA, *F*(1,30)  = 4.26, *p *<* *.05; Figure 5c], whereas D1 receptor and dopamine transporter did not change (data not shown). In contrast, none of these differences was found in the brains of P28 dams (data not shown), consistent with the behavioral effects.

HFD dams exhibited changes in the opposite direction, that is, a significant increase in both the expression of 5‐HTT [significant main effect of HFD, *F*(1,30)  = 4.57, *p *<* *.05; Figure 5b] and of the D2 receptor [significant main effect of HFD, *F*(1,30)  = 5.54, *p *<* *.05; Figure 5c]. P28 HFD dams did not show significant changes in the expression of 5‐HTT or D2 receptor (data not shown).

Next, we employed HPLC to probe changes in the levels of monoamine neurotransmitters in the hippocampus of P8 and P28 dams. We found no significant differences in the levels of 5‐HT, 5‐HIAA (the primary metabolite of serotonin), or the serotonin turnover rate (calculated as 5‐HIAA/5‐HT), in either P8 (Figure 5d) or P28 dams (data not shown). However, we did discover marked decreases due to BCAA in the levels of dopamine metabolites DOPAC [significant main effect of BCAA, *F*(1,24)  = 7.73, *p *<* *.05] and HVA [significant main effect of BCAA, *F*(1,24)  = 13.43, *p *<* *.005; significant HFD x BCAA interaction, *F*(1,24)  = 4.69, *p *<* *.05, due to greatest decrease in LFD/BCAA, post hoc, *p *<* *.05] in the hippocampus of P8 dams. Although total DA content did not differ, the dopamine turnover rates (i.e., DOPAC/DA and HVA/DA), which are a better measure of synaptic dopamine release (Melamed, Hefti, & Wurtman, 1980), were both significantly lower in BCAA dams [DOPAC/DA: significant main effect of BCAA, *F*(1,22)  = 13.27, *p *<* *.005, Figure 5e; HVA/DA: significant main effect of BCAA, *F*(1,22)  = 8.38, *p *<* *.01, Figure 5f]. None of these changes was evident in P28 dams (data not shown), by which time the PPD/A‐like phenotype had disappeared.

We also examined the prefrontal cortex, which receives substantial dopaminergic innervation from striatal regions. We found a similar pattern to the hippocampus, such that BCAA induced a significant decrease in the levels of DOPAC [significant main effect of BCAA, *F*(1,27)  = 14.24, *p *<* *.005, Figure 5h] and HVA [significant main effect of BCAA, *F*(1,27)  = 15.13, *p *<* *.005, Figure 5i] in P8 dams, but not P28 dams (data not shown). There were no significant differences in dopamine turnover rates, due to a similar trend for a decrease in DA itself, although this did not reach significance. There were also no significant differences in serotonergic neurochemistry in this region (Figure 5g).

### 3.5 Western‐type diets and PPD/A are associated with altered metabolomic profiles in both mice and humans

**Figure 6:**
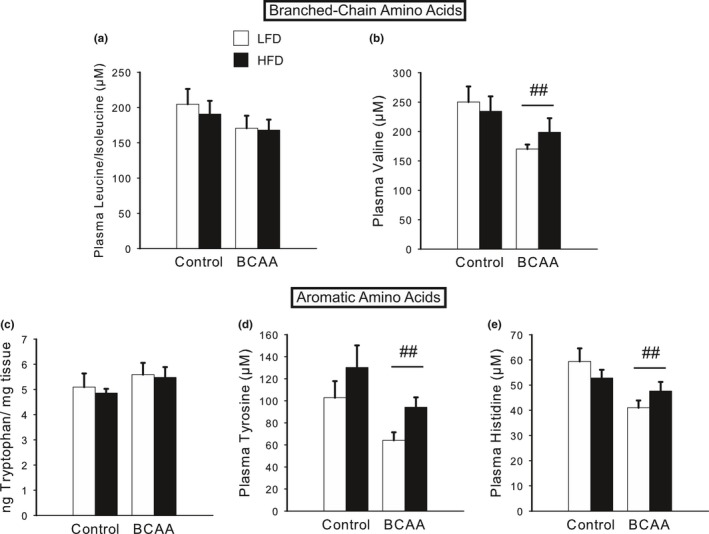
Diets high in BCAA and fat alter metabolomics profiles, particularly neurotransmitter precursors, in postpartum dams. (a) BCAA‐supplemented dams tended to exhibit decreased circulating levels of leucine/isoleucine at P8. (b) Valine was significantly decreased in the plasma of P8 BCAA dams. (c) Tryptophan levels did not significantly differ by diet in the hippocampus of P8 dams. (d) Tyrosine levels in the plasma were significantly decreased by BCAA supplementation at P8, whereas HFD tended to increase circulating levels of tyrosine. (e) P8 BCAA dams exhibited a significant decrease in circulating histidine levels. Data are mean ± SEM,* n *=* *7–8/group. ^##^ *p *<* *.05, BCAA versus Control

One long‐term goal of this body of research is to identify a circulating biomarker that could aid diagnosis of PPD/A, as well as elucidate its mechanistic underpinnings. Thus, we completed a full metabolomic panel of the circulating amino acids, many of which are precursors for key neurotransmitters, in the plasma of P8 dams. To our surprise, the circulating levels of BCAA were decreased in the BCAA‐supplemented dams; specifically, valine was significantly decreased [*F*(1,27)  = 6.38, *p *<* *.05; Figure 6b], whereas leucine/isoleucine did not significantly differ (Figure 6a). Next, we examined the levels of the aromatic amino acids. Tryptophan did not differ in its levels in the hippocampus (Figure 6c) or prefrontal cortex (data not shown), in accordance with the lack of change in serotonin levels. However, tyrosine levels in the plasma were significantly decreased by BCAA [main effect of BCAA, *F*(1,27)  = 6.96, *p *<* *.05; Figure 6d], which is in agreement with the observed decrease in dopamine metabolites and PPD/A‐like phenotype. Histidine, an aromatic amino acid and the precursor for histamine, was also significantly decreased by BCAA [*F*(1,27)  = 9.06, *p *<* *.01; Figure 6e].

We next analyzed data from the human cohort to establish preliminary clinical evidence for the relationships among diet, GWG, BCAA concentrations, and PPD/A identified in the animal studies. The sample included 17 primiparous women between 20–36 years of age (M = 29.24; SD = 5.04) who gave birth to a healthy, full‐term (mean gestational age = 40.1 weeks) infant via cesarean (*N *= 9) or vaginal (*N *= 8) delivery. Prior to pregnancy, mean BMI was 25.99 (SD = 3.53; range = 21.13–34.28) with six normal‐weight women, nine overweight women, and two class I obese women. After pregnancy, mean BMI was 33.72 (SD = 3.27; range = 28.29–38.60), resulting in a mean %ΔBMI of 30.56 (SD = 9.68; range = 12.6–44.1). Mean EPDS scores were 6.76 (SD = 4.69; range 0–18), and using a cut‐off score of 10, 23.5% of the sample (*N *= 4) had significant risk for PPD/A.

**Figure 7:**
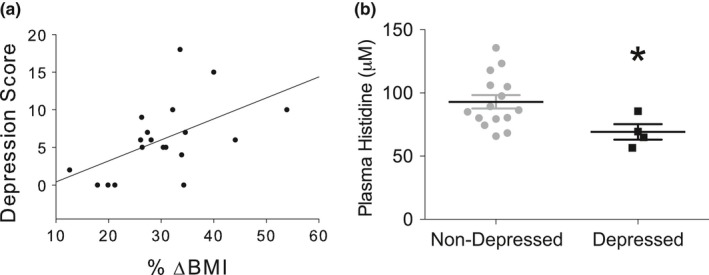
Human studies reveal that gestational weight gain is positively associated with PPD risk, and plasma histidine should be investigated further as a prospective biomarker for PPD. (a) In our cohort of women, % ΔBMI during pregnancy is positively correlated with self‐reported depression (EPDS scores), such that % ΔBMI accounts for 29% of the variance in depression scores. (b) Metabolomics analysis of postpartum human plasma identified histidine as the only amino acid that was significantly changed in women with PPD risk, which is in agreement with our animal model

As it is difficult to control or manipulate diet in humans, we used excess GWG as a proxy for excess caloric intake (Stuebe, Oken, & Gillman, 2009), which we hypothesized to be a risk factor for the development of PPD/A. Accordingly, we analyzed the association between %ΔBMI during pregnancy in our cohort and EPDS scores, and discovered a significant positive correlation [*r*_*s*_(19)  = 0.54, *p *<* *.05; Figure 7a]. This correlation has an R^2^ of 0.29, meaning that the %ΔBMI accounts for 29% of the variance in EPDS scores.

Analysis of plasma amino acids showed that of the 15 amino acids measured, changes were observed only in histidine, where mean levels were significantly lower (Wilcoxon *p *=* *.045) in women with PPD/A risk versus without PPD/A risk (Figure 7b), which is concordant with the findings in mice, as described above.

## 4 DISCUSSION

This study represents the first reported evidence that perinatal consumption of a Western‐type diet high in fat and BCAA, and associated excess GWG, may increase the risk for PPD/A, findings that warrant future investigation to validate the relationship among perinatal diet, PPD/A, and associated mechanisms. In mice, consuming a diet high in BCAA induced a depressive‐like phenotype that was not observed in nonpregnant females, suggesting PPD risk increases as a function of the interaction between pregnancy and components of the Western diet. This phenomenon is further compounded by the addition of high fat, which we and others have shown induces anxiety‐like behavior (Bilbo & Tsang, 2010; Bolton & Bilbo, 2014). Moreover, we report for the first time that this evidence of a relationship between GWG and PPD/A risk identified in mice may extend to humans, although future studies with larger samples sizes should validate these findings. In our study, women who gained more gestational weight as measured by percent change in BMI reported more symptoms of PPD/A. Moreover, we have potentially identified that specific dietary components affect both excess GWG and PPD/A. Should these findings hold true in larger studies, they will have important implications for prenatal care. First, few modifiable risk factors for PPD/A, such as diet, have been established. Second, if dietary components indeed affect GWG and PPD/A, this could lead to modifications in the current standard of care for prenatal dietary counseling, especially for women with a previous history of depression, anxiety, or other risk factors that increase the likelihood of developing PPD/A. High‐risk women may need to be screened for specific dietary components, such as the proportion of red meats and saturated fats consumed, as well as counseled on how these dietary components and associated caloric intake during pregnancy may increase risk for excess GWG and PPD/A.

**Figure 8:**
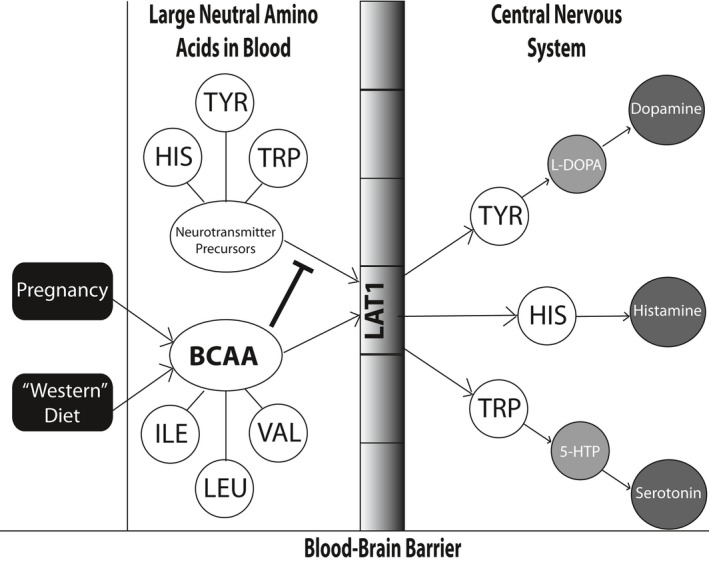
Proposed mechanism for how perinatal consumption of a Western diet may result in increased postpartum depression risk. BCAA levels increase as a function of normal pregnancy and excess dietary consumption, and compete with the amino acid precursors (e.g., histidine, tyrosine, and tryptophan) to mood‐altering neurotransmitters, for transport across the blood–brain barrier via their common transporter, LAT‐1, thereby limiting their uptake. Because synthesis of these neurotransmitters, including histamine, dopamine, and serotonin, is dependent on the availability of their precursor amino acids, increased concentrations of BCAA decrease their production and thus, increase PPD risk

This study is also the first to identify a potential amino acid biomarker for PPD/A risk. Plasma metabolomic profiling showed that levels of histidine, an α‐amino acid, were significantly lower both in the animals fed BCAA‐supplemented diets *and* in the women with PPD/A risk. Histidine is a precursor to histamine, which in the central nervous system modulates food intake as well as regulates other neurotransmitter systems associated with depression, including serotonin, dopamine, and norepinephrine (Hough, 1999; Kano et al., 2004; Nutt, 2008). Tyrosine levels in the animal plasma were also significantly decreased by BCAA. The decreased levels of these two amino acids are concordant with the decreased levels of dopamine metabolites and the PPD/A‐like phenotype identified in the mouse dams. These findings suggest a possible mechanism (see Figure 8) whereby perinatal consumption of a diet high in fats and BCAA, typical of a Western diet, affects the generation of the neurotransmitters that regulate mood (e.g., histamine, dopamine). When availability of BCAA increases as a function of normal pregnancy and excess dietary consumption, there is a subsequent decreased uptake of the amino acid precursors to mood‐altering neurotransmitters, including histamine, dopamine, and serotonin, via their common transporter, LAT‐1, through the blood–brain barrier. Because synthesis of these neurotransmitters is dependent on the availability of their precursor amino acids, increased concentrations of BCAA decrease their production and, thus, increase PPD/A risk.

Contrary to the prediction from our model in Figure 8, we found slightly decreased, or unchanged, levels of circulating BCAA in BCAA‐supplemented mouse dams. However, the regulation of circulating BCAA levels is complex, and plasma levels of BCAA are unlikely to be reliable markers of BCAA status (Tom & Nair, 2006). A decrease in plasma BCAA could indicate increased oxidation, decreased protein breakdown, or increased protein synthesis in the muscle, but this could also indicate that BCAA are being transported across the blood–brain barrier at a higher rate, thus inhibiting the transport of other large neutral amino acids into the brain. We were also surprised to find no clear changes in serotonin levels or metabolites in animals with PPD/A‐like behavior. Rather, there were significant changes in dopamine metabolites in the brain, and circulating tyrosine and histidine. However, the etiology of depression is complex, and in recent years, other studies have found roles for dopamine and histamine in depression and anxiety (Raber, 2005; Tye et al., 2013; Zweifel et al., 2011). Research on the maternal brain in humans has also revealed that new mothers exhibit greater activation of the dopaminergic system of the caudate nucleus in response to the body odor of newborns than nulliparous women (Lundström et al., 2013). Although this study did not investigate maternal–infant interactions among women with PPD/A, others have demonstrated that PPD/A involves impaired mother–infant bonding (O'Higgins, Roberts, Glover, & Taylor, 2013; Tietz, Zietlow, & Reck, 2014), which could relate to decreased activation of reward centers in the brain in response to infant cues. Therefore, our findings may represent novel areas for future research to distinguish PPD/A in women from major depression and generalized anxiety disorder that are not associated with pregnancy, parturition, or postpartum status.

It is notable that our model evoked depressive‐like and anxiety‐like behavior and neurochemical changes in the early postpartum period that dissipated by the end of the lactation period. This time‐dependence lends construct validity to our model, as symptoms of PPD/A in humans typically occur during the first year postpartum (Goodman, 2004; Oʼhara & Swain, 1996). P28 in mice is after the pups will naturally begin to wean themselves and switch to solid food (~P25), so it is at the end of the lactation period (Curley et al., 2009; Hall & Williams, 1983). It is thus possible that the behavioral and neurochemical changes we observed could be linked to lactation, and specifically, that we identified a susceptibility in the BCAA group that is unmasked by lactation. Future studies should explore this possibility.

In our animal model, the observed changes in maternal mood were also associated with alterations in maternal care, which may have serious consequences for offspring health and development. Indeed, PPD/A in humans have been linked to adverse child outcomes, including later cognitive deficits and emotional disturbances (Feldman et al., 2009; Murray & Cooper, 1996; O'CONNOR, Heron, & Glover, 2002), and PPD specifically has been associated with offspring growth retardation and risk for being underweight (Rahman, Iqbal, Bunn, Lovel, & Harrington, 2004). Similarly, we observed that BCAA mothers had smaller pups at weaning. HFD/BCAA dams also had fewer pups survive to weaning, which may be a consequence of poor maternal care and neglect, or may be related to poor health of the pups due to maternal diet, but independent of maternal behavior. Further research is needed to distinguish between these possibilities.

HFD alone in our animal model resulted in multiple indicators of anxiety‐like behavior throughout the perinatal period, including more stretch‐attend postures in the EZM, excessive self‐grooming, and less time immobile in the FST. Less time immobile in the FST indicates increased activity and struggling in response to the stress of the test, in agreement with the observed anxiety‐like behaviors in the EZM and maternal care. Similarly, previous studies have noted an association between increased anxiety‐like behaviors and decreased time immobile in the FST (Detke et al., 1995; Heisler et al., 1998). It is also possible that HFD may be protective against depressive‐like behavior, although future studies are needed to confirm this hypothesis. Excessive self‐grooming, observed in HFD dams, is one of the hallmark symptoms in an OCD mouse model, which is also associated with increased anxiety‐like behaviors (Shmelkov et al., 2010; Welch et al., 2007). Notably, studies in humans have found that up to one third of women report obsessive‐compulsive behaviors in the early postpartum period, often in association with negative maternal mood (Miller, Hoxha, Wisner, & Gossett, 2015).

While the current studies suggest that perinatal dietary intake, and specifically consumption of a Western diet and associated GWG, increases PPD/A risk, additional studies are needed to validate this relationship and the proposed mechanism of action in humans. Controlled feeding trials in pregnant women allow for the most dietary regulation and may provide the data necessary to validate findings from the current animal models. Additionally, behavioral interventions designed to change dietary components during pregnancy (e.g., reduce proportions of unhealthy fats and BCAA/red meats consumed) should be conducted to determine whether altering these components lowers incidence of PPD/A for women with and without other biobehavioral risk factors for PPD/A. Finally, studies that consider the role of genetic risk for mood disorders and obesity in the development of PPD/A should include metabolomic profiling to determine whether associated metabolite patterns may be used as a biomarker for PPD/A risk.

Findings from this study must be considered in light of several limitations. First, the sample size for the human cohort was small, and findings may not be generalizable to all women with PPD/A risk. Second, animal models, although extremely valuable for translational research in many instances, cannot always translate directly to clinical populations (Seok et al., 2013; Takao & Miyakawa, 2015). The model of a Western diet that we utilized enabled us to study the components of high fat and high BCAA separately and in interaction with each other to address our mechanistic hypothesis, but at the cost of including a high carbohydrate or high sugar component, which has also been implicated in a Western‐type diet. Third, we did not have data on prenatal dietary intake or mood among human participants. However, previous studies have shown that excess GWG is associated with total energy consumed prenatally, consumption of fried foods, which are high in both saturated fats and BCAA, and consumption of a Western diet (Stuebe et al., 2009). Moreover, excess GWG was associated with higher EPDS scores, even without consideration of dietary patterns. Thus, the relationship of GWG to PPD/A should be investigated further, including determining whether this relationship is stronger among women with depression history and/or prenatal mood disorders.

Despite these limitations, this is the first study to our knowledge to investigate the effects of excess GWG on PPD/A risk using animal and human data. Our findings suggest this is a fertile area that warrants future research, especially given that diet is a modifiable behavior, and relatively low risk to institute during pregnancy if based on principles of sound nutrition.

## AUTHOR CONTRIBUTIONS

JLB, CK, GS, SDB, and LAS designed the studies; JLB, MGW, BR, ST, MS, DCB, NYY, OI, SWW, CLS, SDB, and LAS performed the research; CN contributed analytic tools; JLB, CLS, TMO, and LAS analyzed data; and JLB, SDB, and LAS wrote the article.

## CONFLICT OF INTEREST

All of the authors reported no biomedical financial interests or potential conflicts of interest.
